# Endoscopic Approach at Two Non-Sequential Levels in Lumbar Discitis

**DOI:** 10.7759/cureus.22158

**Published:** 2022-02-12

**Authors:** Fabio Henrique P Da Silva, Carlos Eduardo P Henriques, Dennis L Moreira, Flavio N Leira, Rubem David D Reis

**Affiliations:** 1 Neurosurgery, Hospital Naval Marcilio Dias, Rio de Janeiro, BRA

**Keywords:** lower back pain, antibiotic usage, lumbar spine surgery, endoscopy, discitis

## Abstract

We report a case of spondylodiscitis in two non-sequential segments of the lumbar spine that was unresponsive to antibiotic treatment instituted and guided by results of blood and urine cultures.

A 70-year-old female was admitted to our hospital with complaints of adynamia, low fever, and severe lower back pain that caused difficulty in mobilizing the lower limbs. Spinal tomography and magnetic resonance imaging (MRI) of the lumbar spine suggested L2L3 and L5S1 spondylodiscitis. After an initial period of improvement, the patient’s condition began to deteriorate again four weeks after initiating the antibiotic therapy. We then opted for surgical treatment through a full-endoscopic transforaminal route, with the aim of collecting intervertebral discs material and performing debridement. After the procedure, the patient experienced immediate relief from the pain and was able to stand and walk without assistance. Cultures from disc fragments showed different bacterial species than that found in the first examination.

The endoscopic approach allowed less tissue damage, debridement of the disc, collection of multiple fragments, thereby facilitating the best antibiotic therapy, and shortening the duration of hospital stay.

## Introduction

The term "spondylodiscitis" refers to the primary infection of the intervertebral disc by a pathogen and secondary osteomyelitis of adjacent endplates, usually occurring in conjunction with each other. In developed countries, the incidence of spondylodiscitis varies between 1:100,000 and 1:250,000. This condition is a major manifestation of hematogenous osteomyelitis in patients over 50 years of age and represents approximately 3%-5% of all osteomyelitis cases [1].

From the point of view of diagnosis, infections involving the intervertebral disc are usually challenging. This is because the onset is often sudden, without any symptoms. In addition, there are difficulties in identifying the causative agent that is necessary for an adequate treatment. A delay in early diagnosis and instituting therapy may lead to instabilities, deformities, sepsis, and, in some cases, even death [2].

Spinal infections can occur spontaneously in patients who are immunocompromised because of the hematogenous dissemination through an infectious focus or following surgical procedures, which has been increasing progressively due to the greater number of procedures performed on the spine. The target for the treatment of spondylodiscitis involves treating the infection, neurological protection, and maintaining the structural alignment of the spine [3].

Approaches for detecting the causative organism include blood and urine culture and acquisition of image-guided tissue. However, the rate of identifying the causative pathogen is reported to be variable [4].

This study aimed to report a case of spondylodiscitis in two non-sequential segments of the lumbar spine using a full-endoscopy technique, which facilitated the identification of the causative agent without causing any harm to the lumbar spine stability.

## Case presentation

A 70-year-old female was admitted with complaints of adynamia, low fever, severe lower back pain, and difficulty in mobilizing the lower limbs due to the associated pain. On clinical examination, she was pale, hypohydrated, with a low fever, and in an antalgic position due to the severe pain. She was unable to adopt a standing or sitting position because of the severe intensity of the lumbar pain. The patient reported having chronic renal failure, non-dialysis, systemic arterial hypertension, and diabetes mellitus.

Laboratory tests on admission revealed leukocytosis and elevated C-reactive protein (CRP) levels. Blood and urine samples were collected, and antibiotic treatment with vancomycin (500mg IV every 12 hours) was started empirically.

**Figure 1:**
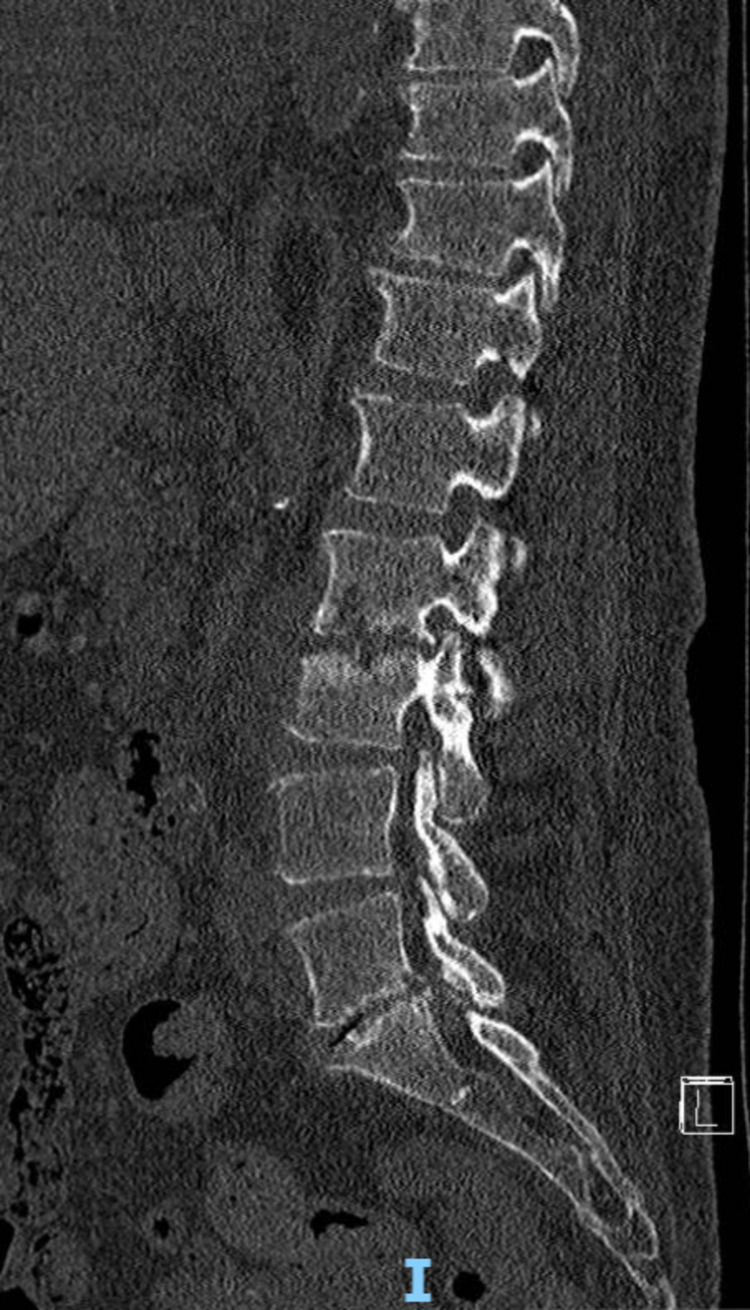
Sagittal CT view of the lumbar spine with changes in L2L3 and L5S1 endplates

**Figure 2:**
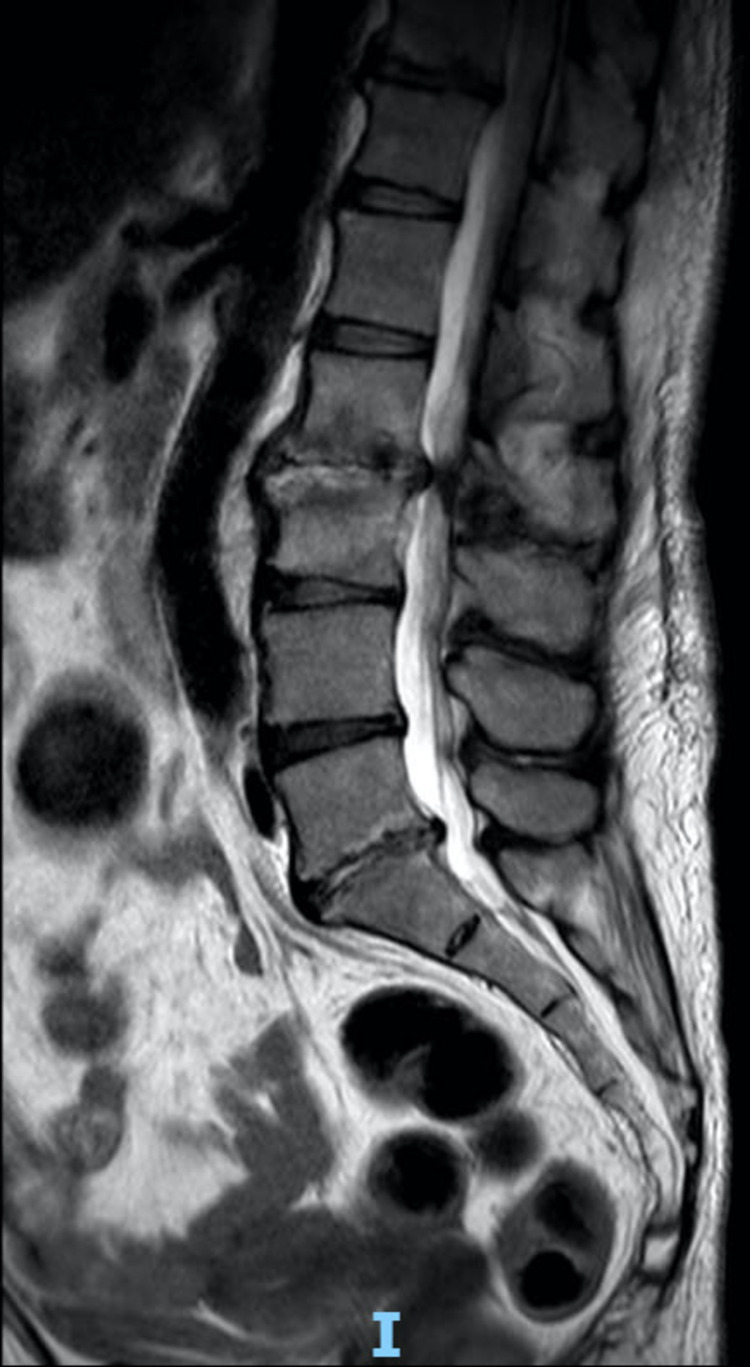
Sagittal MRI view of the spine (T2-weighted) with a hypersignal in the L2L3 and L5S1 disc spaces

She underwent spinal tomography (Figure 1) and magnetic resonance imaging (MRI) (Figure 2) of the lumbar spine, which were suggestive of L2L3 and L5S1 spondylodiscitis. As the blood and urine samples showed the presence of *Enterococcus faecalis*, she was started with meropenem (1g IV every 12 hours) for four weeks initially, according to clinical and laboratory response. Though the antibiotics along with hyperbaric oxygen therapy initially improved the infectious condition, the low back pain persisted. After an initial period of improvement, she developed a low-grade fever and worsening of laboratory parameters despite four weeks of antibiotic therapy. After discussing the case, an endoscopic approach was indicated at both lumbar levels to collect tissue and clean the disc space.

**Figure 3:**
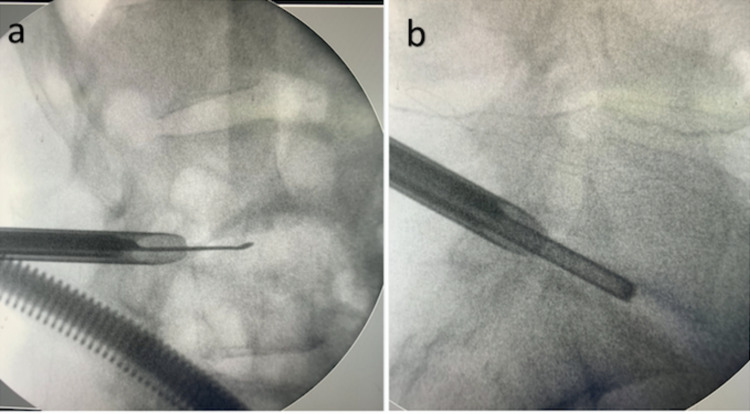
Lateral view of intraoperative X-ray of discs L2L3 (a) and L5S1 (b)

**Figure 4:**
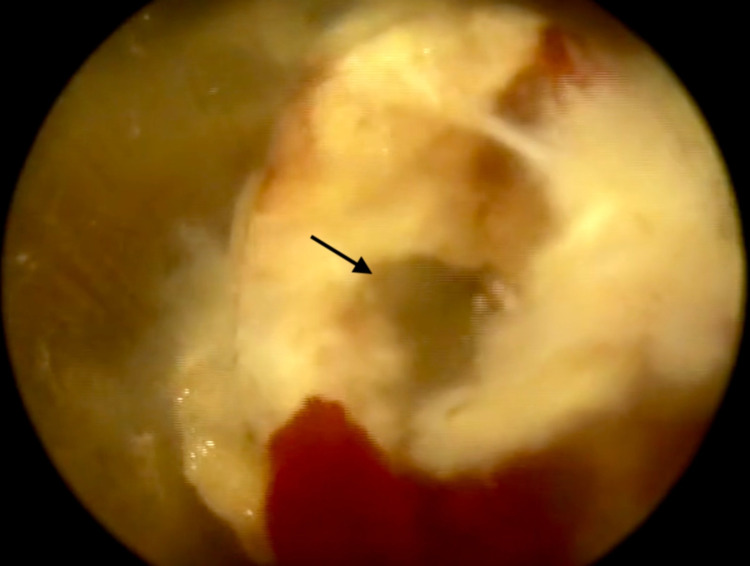
Intraoperative endoscopic view with visualization of collection within the disc space (black arrow)

The patient underwent a transforaminal approach, with an inside-out technique, under local anesthesia and conscious sedation in the prone position. A spinal endoscope (Richard Wolf GmbH, Knittlingen, Germany) with a 30-degree optic was used for the entry point to the L2L3 space approximately 8 cm from the midline and to the L5S1 space 10 cm from the midline, and a spinal needle was inserted into the target disc through Kambin’s triangle (Figure 3). During the procedure, it was possible to identify a purulent intradiscal debri at both levels. Following collection of representative material, the discs were thoroughly debrided with physiological saline solution (Figure 4).

After the surgical procedure, the patient presented immediate relief from the pain, probably by reduction of nociceptive stimulus and resolution of spinal canal stenosis and the compression of spinal roots, and was able to stand and walk without any assistance. Table 1 describes the levels of pain and C-reaction proteins from the initial admission period up to the discharge. The culture of disc fragments identified bacterial species *Stenotrophomonas maltophilia*, which was different from that isolated in the first examination, and the patient was started on trimethoprim/sulfamethoxazole (400mg IV every 12 hours). She was discharged from the hospital after six weeks of treatment with preserved functional capacity. She returned for a follow-up of 30 days, with no complaints of pain or walking without assistance.

**Table 1:** Levels of pain and CRP from the admission period up to discharge AVS, analog visual scale; CRP, C-reactive protein CRP reference value: <1.0mg/dL

Time	AVS	CRP
Admission	10/10	35 mg/dL
21 days	8/10	25mg/dL
40 days	10/10	30mg/dL
Post-operative	4/10	15mg/dL
30 days after discharge	1/10	<1.0mg/dL

## Discussion

Spondylodiscitis is an uncommon disease, and its diagnosis is often difficult due to non-specific initial signs and symptoms. This leads to a delay in diagnosis and subsequently in treatment too [1].

The treatment of spondylodiscitis aims to cure the infectious condition, restore and preserve the structure and stability of the spine, facilitate recovery from neurological deficits, and direct appropriate pain therapy [1].

A number of approaches such as conservative treatment, traditional open surgery, and minimally invasive surgery exist for treating spontaneous spondylodiscitis. Traditionally, the conservative approach comprising the use of broad-spectrum antibiotics and bed rest was considered adequate for the treatment [5]. In addition, routine monitoring of CRP and erythrocyte sedimentation rate, which are mandatory until normality returns or up to two weeks after symptoms improve, is recommended [6].

Hyperbaric oxygen therapy comprises the administration of 100% oxygen at pressures greater than that of the atmosphere and is a recognized treatment for a number of disorders, such as gas gangrene, severe necrotizing soft tissue infections, and chronic refractory osteomyelitis [7]. This therapy is widely used in our institution as an adjuvant in cases of uncomplicated spondylodiscitis.

Though open surgery (anterior, posterior, or both) with or without instrumentation have been documented to yield good results, they are often associated with relatively high complication rates [8].

As shown by Wu et al., and recently in infectious processes, without severe neurological symptoms [2,9-11], endoscopic treatment for uncomplicated disc diseases, which began in the 1980s, has vastly advanced the treatment approach to degenerative diseases.

In the present case, due to the need for approaching two non-sequential levels, endoscopy was chosen. This was because the procedure is performed routinely in our clinical, is minimally invasive, and permits the possibility of performing an exhaustive cleaning of the disc space and structural maintenance of the lumbar spine.

Ito et al. used the posterolateral endoscopic technique on 15 patients with pyogenic spondylodiscitis in the thoracic or lumbar spine. The authors reported that all patients had immediate pain reduction after the surgery, and infections were successfully treated. They concluded that endoscopic debridement via posterolateral and continuous irrigation results in satisfactory clinical outcomes in patients with comorbidities and pyogenic spondylodiscitis [9].

In another study that included 60 patients with spondylodiscitis treated with an endoscopic procedure, all patients experienced rapid pain relief after surgery. Furthermore, there were no changes in sagittal alignment, and pathogens were identified in around 77.27% of patients from samples collected before the use of antibiotics. CRP and erythrocyte sedimentation rate values significantly decreased three months after the endoscopic surgery. Two patients in the series required a new endoscopic approach, and no complications related to the procedure were reported [3].

## Conclusions

Spondylodiscitis at two non-adjacent levels can be a challenge for surgical treatment, as it requires greater exposure to approach both levels. Access to disc infections by endoscopy via the transforaminal route offers the possibility of a direct approach to the disc space, with several advantages such as minimal tissue damage, possibility of using local anesthesia and conscious sedation (required for patients with multiple comorbidities), and greater accuracy in identifying the causative pathogen, without modifying the posterior structures of the spine.
